# Systematic Analysis of Kelch Repeat F-box (KFB) Protein Family and Identification of Phenolic Acid Regulation Members in *Salvia miltiorrhiza* Bunge

**DOI:** 10.3390/genes11050557

**Published:** 2020-05-16

**Authors:** Haizheng Yu, Mengdan Jiang, Bingcong Xing, Lijun Liang, Bingxue Zhang, Zongsuo Liang

**Affiliations:** 1 Institute of Soil and Water Conservation, Chinese Academy of Sciences & Ministry of Water Resource, Yangling 712100, China; yuhz@nwafu.edu.cn (H.Y.); xingbingcong@163.com (B.X.); llj@zafu.edu.cn (L.L.); zhangbx@nwafu.edu.cn (B.Z.); 2 University of the Chinese Academy of Sciences, Beijing 100049, China; 3 Zhejiang Province Key Laboratory of Plant Secondary Metabolism and Regulation, College of Life Sciences and Medicine, Zhejiang Sci-Tech University, Hangzhou 310018, China; jiangmd0729@163.com

**Keywords:** *S. miltiorrhiza*, ubiquitin–proteasome system, KFB protein, secondary metabolite, regulation

## Abstract

*S. miltiorrhiza* is a well-known Chinese herb for the clinical treatment of cardiovascular and cerebrovascular diseases. Tanshinones and phenolic acids are the major secondary metabolites and significant pharmacological constituents of this plant. Kelch repeat F-box (KFB) proteins play important roles in plant secondary metabolism, but their regulation mechanism in *S. miltiorrhiza* has not been characterized. In this study, we systematically characterized the *S. miltiorrhiza* KFB gene family. In total, 31 *SmKFB* genes were isolated from *S. miltiorrhiza*. Phylogenetic analysis of those *SmKFBs* indicated that 31 *SmKFBs* can be divided into four groups. Thereinto, five *SmKFBs* (*SmKFB1*, *2*, *3*, *5*, and *28*) shared high homology with other plant KFBs which have been described to be regulators of secondary metabolism. The expression profile of *SmKFBs* under methyl jasmonate (MeJA) treatment deciphered that six *SmKFBs* (*SmKFB1*, *2*, *5*, *6*, *11*, and *15*) were significantly downregulated, and two *SmKFBs* (*SmKFB22* and *31*) were significantly upregulated. Tissue-specific expression analysis found that four *SmKFBs* (*SmKFB4*, *11*, *16*, and *17*) were expressed preferentially in aerial tissues, while two *SmKFBs* (*SmKFB5, 25*) were predominantly expressed in roots. Through a systematic analysis, we speculated that *SmKFB1*, *2*, and *5* are potentially involved in phenolic acids biosynthesis.

## 1. Introduction

*S. miltiorrhiza*, which belongs to the family Labiate, is a well-known Chinese herb. The dried roots and rhizomes of this plant are known as Danshen in The Pharmacopoeia of The People’s Republic of China, and they are widely used in the clinical treatment of cardiovascular and cerebrovascular diseases in China and, to a lesser degree, in Japan, The United States, and other European countries [1]. Phenolic acids and tanshinones are major pharmaceutical components and secondary metabolites in *S. miltiorrhiza*. There are more than 40 lipophilic tanshinones (tanshinone I, tanshinone IIA, cryptotanshinone, dihydrotanshinone I, and so on), and 20 hydrophilic phenolic acids (salvianolic acid B, rosmarinic acid, dihydroxyphenyllactic acid, and lithospermic acid) have been identified and/or isolated from *S. miltiorrhiza* [2]. In China, many pharmaceutical dosage forms of Danshen are commercially available, such as dripping pills, tablets, injectables, oral liquids, and granules. Among all the available dosage forms, the Fufang Danshen dripping pill and Fufang Danshen tablets are the two most widely used in China. Notably, the Danshen dripping pill has entered into the healthcare market of the United States, Netherlands, Britain, South Korea, and Russia (www.tasly.com). Not only that, but the Fufang Danshen dripping pill was the first Chinese traditional medicine approved by the Food and Drug Administration (FDA) for clinical tests, and it has now completed Phase III clinical trials [3]. 

Due to good curative effect of Danshen, market demand has been increasing in recent years. This requires us to improve the production of tanshinones and phenolic acids via modern biotechnology. Completion of genomic sequencing of *S. miltiorrhiza* [4,5] has provided comparatively perfect information about the genes involved in the tanshinones and phenolic acids biosynthetic pathway [6,7]. Many transcription factors (TFs), such as WRKY, myeloblastosis (MYB), the basic helix–loop–helix (bHLH), and ethylene response factor (ERF), acting as positive or negative regulators, and modulating transcription of biosynthetic enzymes, have been reported [8,9,10,11,12,13,14,15] in recent years. However, less is known about the multifaceted regulatory mechanism controlling tanshinones and phenolic acids biosynthesis beyond the transcriptional level.

The ubiquitination-26s proteasome system (UPS) is one of protein post-translational modifying manners and is involved in modulating nearly all aspects of plant biological processes, including cell division, plant growth and development, hormone signaling, response to both abiotic and biotic stresses, and secondary metabolism regulation [16,17]. UPS begins with the sequential action of three enzymes, E1 (ubiquitin (Ub)-activating enzyme), E2 (Ub-conjugating enzyme), and Ub ligases (E3). Ubiquitin is activated by E1 in an ATP-dependent manner and conjugated to E2. The Ub-E2 intermediate then transfers Ub to a Lys residue of the substrate protein via the E3 recognition element. The ubiquitinated proteins are recognized and degraded by the 26s proteasome [18]. Among these three enzyme families, the most important are the E3s that are in charge of substrate specificity. A total of over one thousand E3s have been identified so far from the genomes of different plant species, which are classified into different families on the basis of their mode of action and subunit composition [19]. One of the best characterized E3s in plants are the Skp1-Cullin-F-box (SCF) protein complexes [20]. SCF E3 complex consists of S-phase kinase-associated protein 1 (SKP1), Cullin 1 (CUL1), RING-box1 (RBX1), and an F-box protein [20]. Among these components, the F-box protein charges the specificity of the SCF complex by selective recruitment of target proteins through the protein–protein interaction domain [21].

F-box proteins constitute a large family in plants and are characterized by a conserved F-box motif (approximately 40~50 amino acids) at their N-terminus, which interacts with Skp1. The C-terminus of F-box proteins generally contains one of several highly variable protein interaction domains, such as the WD40 repeat, tetratricopeptide repeat (TPR), leucine-rich repeat (LRR), armadillo (Arm), jumonji (Jmj)—C domains, Tub, actin, DEAD-like helicase, and the Kelch repeat that interacts with specific protein substrates via UPS degradation [22]. The F-box protein family is further divided into several subfamilies based on a different protein–protein interaction domain at the C-terminus. In this study, we focused on the F-box protein which contains several Kelch motifs at C-terminus, called Kelch repeat F-box (KFB) proteins. Several KFB proteins have been characterized to be regulators of plant secondary metabolism. For example, AtKFB01, AtKFB20, AtKFB50, AtKFB39, and AtKFB^CHS^ participate in the regulation of the phenylpropanoid biosynthetic pathway of *Arabidopsis thaliana* [23,24,25]. *CmKFB* is identified as a negative regulator of naringenin chalcone biosynthesis [26]. In rice, Borah et al. have found that *OsFBK1* affects anther and root secondary cell wall lignin biosynthesis by mediating turnover of cinnamoyl-CoA reductase (CCR), which is the first committing enzyme in lignin biosynthesis [27]. All of above cases show that KFB proteins play an important role in phenylpropanoid biosynthesis by mediating corresponding enzyme degradation. Plant genomes contain a large number of *KFB* genes. For example, *A. thaliana* and *Populus trichocarpa* contain at least 103 and 68 *KFB* genes, respectively [28]. However, little information is available for illuminating *KFB* genes of *S. miltiorrhiza*. 

## 2. Materials and Methods 

### 2.1. Identification and Cloning of SmKFB Gene Family Members

The most recent annotated version of cDNAs from *S. miltiorrhiza* were downloaded from the respective genome and transcriptome sequence sites [4,5,29]. Published *A. thaliana* KFB protein sequences [28] were used as first queries for BLAST searches against those *S. miltiorrhiza* databases. The sequences were selected as candidate sequences for further study if their E value was ≤ e^−10^. Candidate sequences were confirmed for presence of F-box and Kelch domains by use of the HMMER 3.3 [30] and verified via the online Tool of Conserved Domains [31] and SMART [32]. The KFB-like sequences confirmed by HMMER search in the *S. miltiorrhiza* database were in turn used reiteratively to search the *SmKFBs* until no new sequences were found. The KFB sequences in different databases were blasted using Cluster 3.0 software to remove repeat sequences. Full-length open reading frames (ORFs) of *SmKFBs* were amplified by PCR using the primers listed in Supplementary Table S1. The primers were designed using Premier 5.0 software. The cDNA obtained from *S. miltiorrhiza* roots or flowers were used as the template for the gene clone. KOD-Plus-Neo polymerase (TOYOBO, Osaka, Japan) was used for the PCR reaction. The PCR reaction was performed in the Mastercycler^®^ Nexus PCR (Eppendorf, Hamburg, Germany) for 2 min at 94 °C, then for 30 cycles for 10 s at 98 °C, 30 s at 58 °C, and then 60 s at 68 °C. After the final cycle, the amplification was extended for 5 min at 68 °C. The PCR products were gel-purified using the MiniBEST Agarose Gel DNA Extraction Kit (Takara, Dalian, China), ligated to the pClone007 Blunt Vector (TsingKe, Beijing, China), transformed to *Escherichia coli* DH5α, and then sequenced (TsingKe company, Hangzhou, China). The theoretical isoelectric point (pI) and molecular weight (Mw) of the full-length coding sequence (CDS) of *SmKFBs* were predicted using the Compute pI/Mw tool in the BioEdit software (version 7.0.5.3, Sydney, Australia). 

### 2.2. Multiple Sequence Alignment, Phylogenetic Analysis

Multiple sequence alignment of the KFBs from *S. miltiorrhiza*, *A. thaliana*, and *Oryza sativa*, which were performed using CLUSTALW, and phylogenetic trees were constructed via the neighbor-joining method (bootstrap test was replicated 1000 times) using MEGA 6.0 software [33]. All the *SmKFBs* were analyzed by MEME online software [34] for conversed motif prediction with the following criteria: 20 motifs, with an optimum motif width between 8 and 50 residues, with any number of repetitions. The sequences for phylogenetic trees and conversed motif analysis were shown in Supplementary Table S2.

### 2.3. Plant Materials and Growth Conditions

The plants of *S. miltiorrhiza* were grown in the field of the Zhejiang Sci-Tech University Medicinal Herb Garden for 2 years. The roots, stems, leaves, and flowers were collected in May, 2019. The fresh organs were frozen in liquid nitrogen and stored at −80 °C until use. The hairy roots culture system of *S. miltiorrhiza* was referred to by Xing et al. [35]. Samples of the fresh hairy roots of *S. miltiorrhiza* weighing 0.2 g were inoculated into a 100 mL triangular flask containing 50 mL of hormone-free 6, 7-V liquid medium. The triangular flasks containing the hairy roots were the placed in an orbital shaker at 110 rpm and incubated at 25 °C in the dark [36]. Methyl jasmonate (MeJA, Sigma-Aldrich, St. Louis, MO, USA) was diluted with ethanol to a concentration of about 100 mM, and then sterilized through 0.22 μm filters. The MeJA treatment was performed on the 18th day after inoculation. The MeJA was added to the medium to make the final MeJA concentration about 100 μM. Hairy roots treated with pure ethanol were designated as the control. Hairy roots were sampled at 0, 1, 2, 4, 8, 12, and 24 h after treatment. The fresh hairy roots were immediately frozen in liquid nitrogen and stored at −80 °C for RNA extraction.

### 2.4. Transcriptional Analysis by Real-Time Quantitative PCR (RT-qPCR)

Total RNA was extracted from liquid stored sample of *S. miltiorrhiza* using the RNAprep pure Plant Kit (Tiangen, Beijing, China). and then reversely transcribed according to the manufacturer’s instruction of PrimeScript^TM^ RT reagent Kit (Takara, Dalian, China). RNA integrity was analyzed on 1.0% agarose gel. RNA quantity was determined using a NanoDrop 2000 Spectrophotometer (Thermo Scientific, Woburn, USA). The obtained cDNA was used as a template for the RT-qPCR analysis using the QuantStudio^TM^ Flex6 System (ABI, Alexandria, America) with SYBR^®^ green reagents (Takara, Dalian, China). The primers are listed in Supplementary Table S3. *SmUBQ10* was used as an internal control [8]. The RT-qPCR reaction for target gene transcript amplification was carried out in a final volume of 20 μL containing 10 μL TB Green Premix Ex Taq (Takara, Dalian, China), 0.4 μM each forward and reverse primers, and 2 μL (about 100 ng) diluted cDNA. RT-qPCR was performed according to the following conditions: 30 s predenaturation at 95 °C, and 40 cycles for 5 s at 95 °C and 30 s at 58 °C. Experiments were performed in triplicate for each bio-repeat and tech-repeat, and the results were represented by their means ± SD. Quantification of gene expression was done with the 2^−ddCt^ method [37].

## 3. Results

### 3.1. Molecular Cloning of 31 SmKFB Genes from S. Miltiorrhiza

A total of 103 *AtKFB* genes have been identified from *Arabidopsis* genome. To identify the *S. miltiorrhiza SmKFB* gene, *AtKFB* sequences were used as queries to blast against the current assembly of the *S. miltiorrhiza* genome. A total of 49 genes were predicted for *SmKFBs*. Then, we submitted those putative *SmKFBs* to HMMER 3.0, the online Tool of Conserved Domains, and SMART for further analysis. Among 49 putative *SmKFB*s, F-box or Kelch motif were absent in 18 genes. Hence, 31 *SmKFBs* were used for further analysis. To verify the predicted gene models and correct the errors of sequences, PCR amplification was done on the CDS (coding sequence) of all 31 *SmKFB*s genes using the primers listed in Supplementary Table S1, and then cloned and sequenced. As shown in Table 1, the sequence analysis of 31 identified *SmKFB* genes demonstrated deduced amino acid (AA) numbers from 342 to 478. *SmKFB* showed a wide range of isoelectric points (pI) from 4.76 to basic 9.72, indicating extensive distribution in different subcellular areas. Molecular weights (Mw) of these proteins ranged from 37898.69 to 54239.94 Da. Among the 31 *SmKFBs*, 11 contain a single Kelch motif, six have two Kelch motifs, 12 have three Kelch motifs, one has four Kelch motifs, and the remaining one has five Kelch motifs. The different number of Kelch motifs in *SmKFB* proteins indicate that those *SmKFBs* may interact with a variety of proteins to regulate the physiological process.

### 3.2. Phylogenetic Analysis and Conserved Motifs Identification of the S. Miltiorrhiza KFB Family

**Figure 1:**
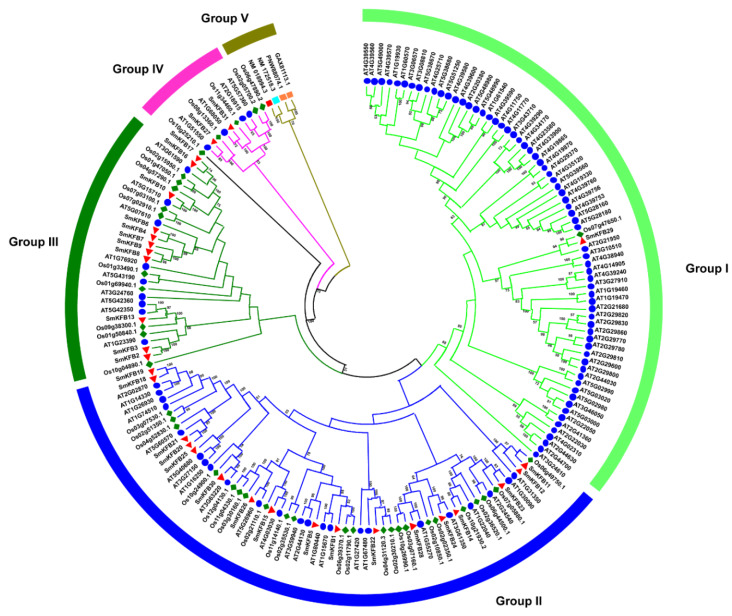
Phylogenetic analysis of Kelch repeat F-box (KFB) proteins from *S. miltiorrhiza* and other organisms. The tree was constructed from amino sequences using MEGA 6.0 via the neighbor-joining (NJ) method with 1000 bootstrap replicated. The blue circles, red triangles, dark green diamonds, red squares, orange pale squares, and blue squares represent KFBs from *A. thaliana*, *S. miltiorrhiza*, *O. sativa*, *Homo sapiens*, *M. musculus*, and *C. reinhardtii*, respectively. Clades with different colors represent diverse groups.

To get a detailed knowledge of the evolutionary relationship and topological structure of the *S. miltiorrhiza* KFB protein family, a neighbor-joining (NJ) phylogenetic tree was constructed from 31 *S. miltiorrhiza* KFB proteins, 103 *A. thaliana* KFB proteins, 46 *O. sativa* KFB proteins, two *Chlamydomonas reinhardtii* KFB proteins, one *Homo sapiens* KFB protein, and one *Mus musculus* KFB protein. All plant KFB protein clades were separated from animal KFB clades (Figure 1). Due to the absence of KFB protein in Charophyceaes, a small group of predominantly freshwater green algae represents the most recent common ancestor of land plants [27]; we rooted the tree with human KFB, *M. musculus* KFB, and two *C. reinhardtii* KFB proteins. According to the well-supported bootstrap data, the phylogenetic tree divided the KFBs into five clades, named from Groups I to V.

Group I was the largest group with 69 plant KFB proteins, of which, 67 were from *A. thaliana*. The result is consistent with previous reports showing that *A. thaliana* KFB proteins have been expanded when various eudicot species diverged from their respective most recent common ancestor [27]. Group II, III, and IV KFB proteins did not reflect any species specificity, as their KFB proteins have orthologs in other analyzed species. It is conceivable that those KFBs may perform functions in developmental or physiological processes conserved in land plants. Group IV is the second smallest group after Group V, consisting of 10 plant KFB proteins, which might resemble the ancestral plant KFB. There are two reasons for supporting this idea. Firstly, phylogenetic results indicate that Group IV is phylogenetically closed with Group V, which is the root clade of KFBs. Secondly, AT1G68050/FKF1, AT2G18925/LKP2, and AT5G57360/ZTL proteins contain the N-terminal light–oxygen–voltage (LOV) motif, which was also identified in several proteins of archaea, eubacteria, and eukaryotes [38]. The LOV motif binds to flavin mononucleotide (FMN) to constitute blue light sensors. The blue light photoreceptor is the first and originating photoreceptor system found in all kingdoms of life [39,40], further supporting the idea that Group IV might be the ancestral proteins, and could be important in the evolutionary history of plants from eukaryotic algae to towering trees.

**Figure 2:**
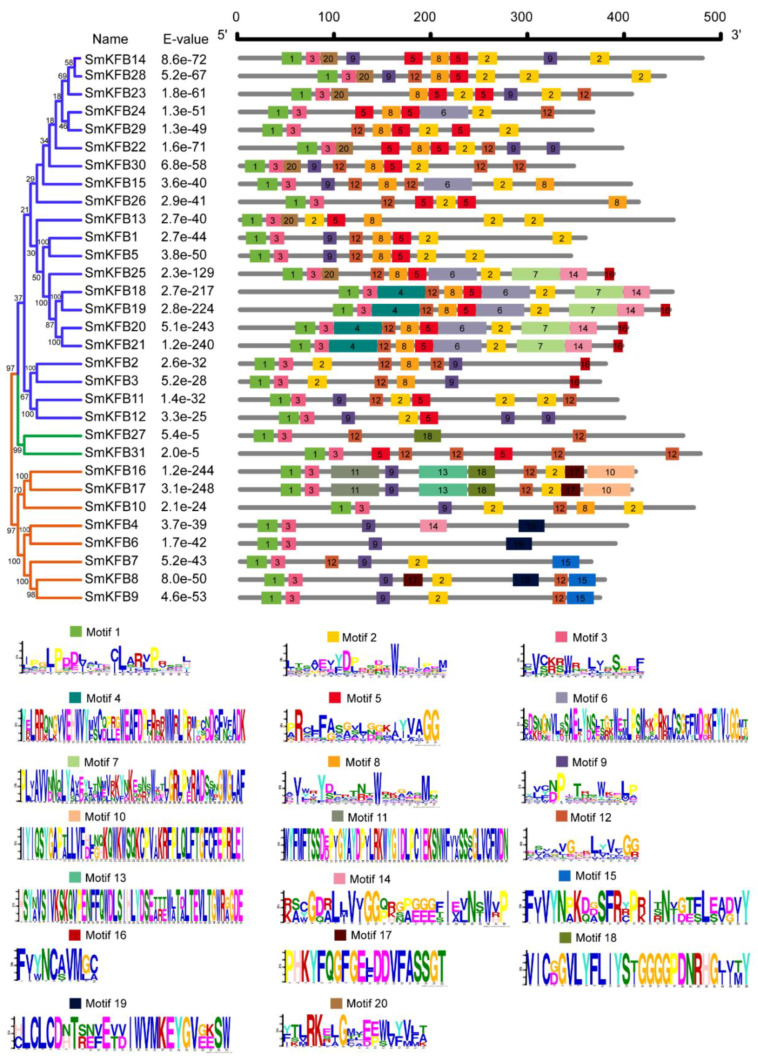
Schematic diagram of conserved motifs in *SmKFB* proteins of *S. miltiorrhiza***.** The different colored boxes represent different motifs and the box size indicates the length of the motif. Sequence logos are shown on the base side of the figure.

In order to understand the similarity and diversity motif of *SmKFBs* within the same group, 31 *SmKFB* amino acid sequences were analyzed by MEGA6.0 for phylogenetic tree construction, and MEME online software was used to predicate the conserved motif. There were 20 distinct motifs which were identified. As shown in Figure 2, most of the *SmKFBs* in the same group had similar motif composition and some motifs were absent in all sequences. For example, motifs 1 and 3 were shared by each group, while some motifs (4, 6, 7, and so on) were only distributed in a specific group. In addition, motif 2 was usually distributed more than once in some sequences. 

### 3.3. Differential Expression of SmKFB Genes in Response to MeJA

Jasmonic acid, and its derivative methyl jasmonate (MeJA), are collectively known as jasmonates (JAs). JAs can serve as a key elicitor in regulation of a wide array of secondary metabolites, including terpenoids, phenylpropanoids, and alkaloids [41,42]. Hence, JA-responsive genes may be a regulator of secondary metabolism. In order to test whether *SmKFBs* were responsive to MeJA treatment in *S. miltiorrhiza*, the expression level of *SmKFBs* in hair root treated with MeJA was analyzed using the RT-PCR method.

**Figure 3:**
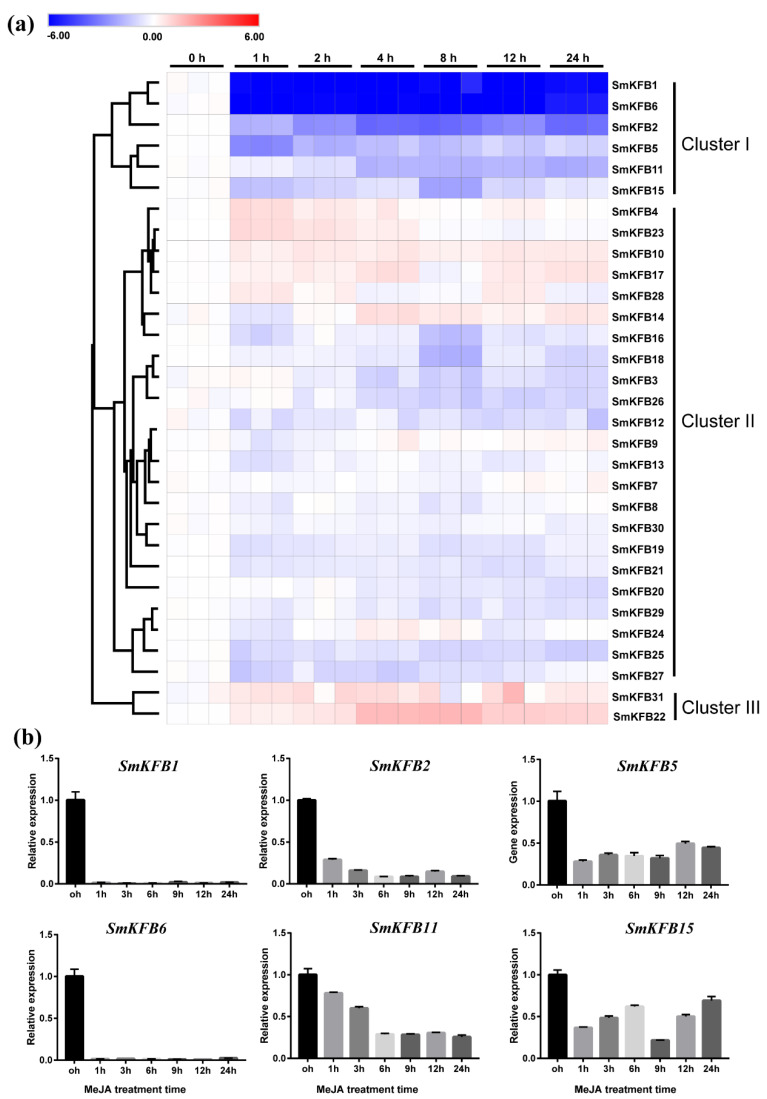
Expression patterns of 31 *SmKFB* genes in hair roots treated with methyl jasmonate (MeJA). (**a**) The gene expression level was detected via RT-qPCR. The transcripts at the 0 h time point were used as control. Fold change in transcript abundance is illustrated as a heat map on a natural log scale. Blocks with colors indicate low/down expression (blue), high/up expression (red), and non-expression/no change (white). (**b**) Expression patterns of six selected genes in response to MeJA.

As shown in Figure 3a, all of the 31 *SmKFBs* having expression profiles were roughly clustered into three groups based on their expression pattern. Of the three groups, the genes in Cluster I were mainly downregulated after MeJA treatment. For example, *SmKFB1*, *SmKFB2*, and *SmKFB6* were markedly downregulated by MeJA treatment with a similar expression pattern (Figure 3b). The gene expression of *SmKFB5* decreased by about 2 times with the MeJA treatment. In addition, the gene expression pattern of *SmKFB11* was slowly downregulated along with the sample points. However, the transcript level of *SmKFB15* was slowly upregulated within 3 h after treatment. 

In Cluster II, 23 *SmKFB* transcripts were probably insensitive to MeJA. Detailed gene expression information is available in Supplementary Table S4. As shown in Figure 3a, members of this group were divided into: (1) the slightly upregulated subgroup; and (2) the slightly downregulated subgroup. Six *SmKFB* genes (*SmKFB4, 10, 14, 17, 23, and 28*) were slightly upregulated, while 17 were downregulated by MeJA treatment at the sampling time point. In Cluster III, the gene expression of *SmKFB22* and *SmKFB31* was upregulated by MeJA, suggesting their potential participation in the degradation of some repressed proteins, which blocked JA signal transduction.

### 3.4. Tissue-Specific Expression of SmKFBs

**Figure 4:**
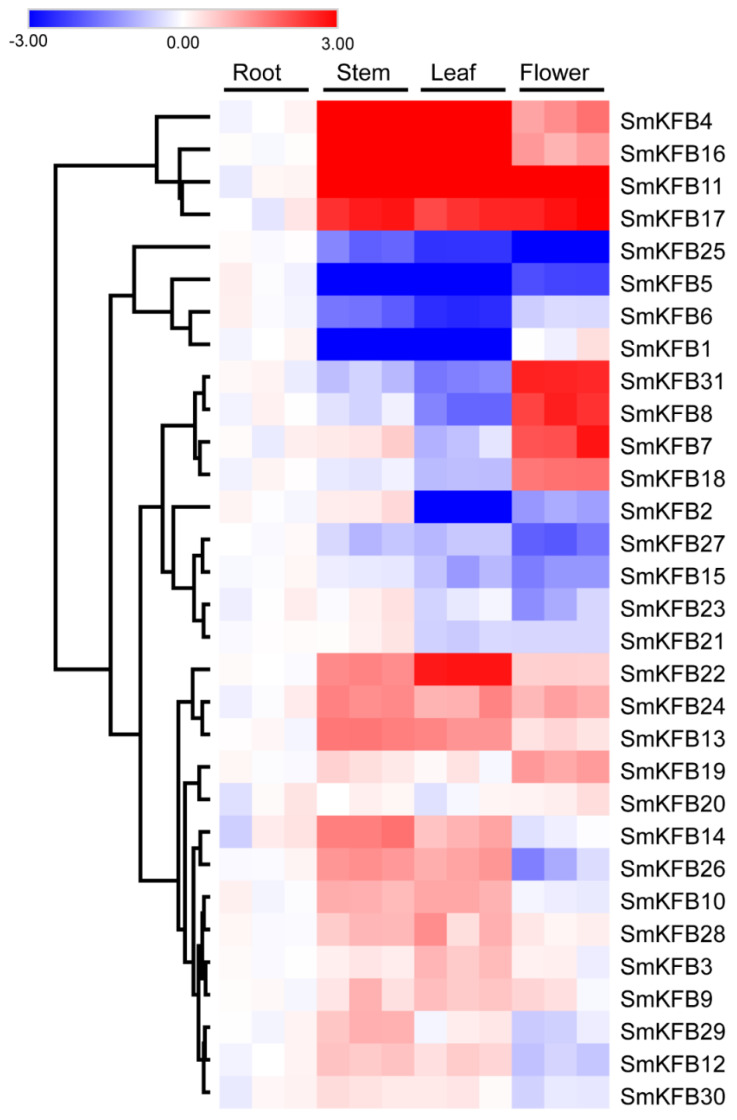
Expression of *SmKFB* genes in different tissues of the *S. miltiorrhiza* plant. The transcript levels in roots were used as control. The blue block indicates lower expression than root, and the red block represents higher expression than root. The color depth of blocks indicates the transcript abundance.

Tanshinones and phenolic acids are mainly distributed in the root of *S. miltiorrhiza*. However, tanshinones were not detected in the aerial parts of *S. miltiorrhiza* [43]. There was an uneven distribution of tanshinones and phenolic acids in *S. miltiorrhiza* tissue, indicating those secondary metabolism related genes have tissue-specific gene expression. Therefore, in this study, we examined 31 *SmKFBs* gene profiles from different tissues of this plant. As shown in Figure 4, all of the 31 *SmKFBs* genes were expressed in all analyzed tissue but with a differential expression pattern. 

Four *SKFBs* (*SmKFB4*, *11*, *16*, and *17*) showed predominant expression in all the aerial tissues. Four *SmKFBs* (*SmKFB7*, *8*, *18*, *31*) showed only higher expression in flowers. Eleven *SmKFBs* (*SmKFB3*, *9*, *10*, *12*, *13*, *14*, *22*, *24*, *26*, *28*, *29*) were mainly expressed in the stem and leaf, while *SmKFB1* and *SmKFB6* were expressed at a lower level in the stem and leaf. The other nine *SmKFBs* showed a slight change in all analyzed tissues, suggesting these genes are likely to play a ubiquitous role in *S. miltiorrhiza*. Some phylogenetically closed *SmKFBs* showed different expression patterns. For example, *SmKFB2* and *SmKFB3* were closely related in the phylogenetical tree. However, *SmKFB2* was expressed at lower levels in leaf, while *SmKFB3* showed higher expression levels in stem, indicating that *SmKFBs* within a common phylogenetic clade can be differentially regulated at the mRNA level, and therefore independently mediate the same physiological process under different conditions. 

### 3.5. Identification of Candidate SmKFB Genes Related to Phenolic Acid Biosynthesis

It has been reported that several plant KFBs were identified to function as regulators of phenylpropanoid biosynthesis. For example, six KFB proteins form *A. thaliana* (KFB01/AT1G15670, KFB20/AT1G80440, KFB39/AT2G44130, KFB50/AT3G59940, KFB07/AT1G23390, and SAGL1/AT1G55270)-regulated phenylpropanoid or flavonoid biosynthetic pathways by controlling the degradation of phenylalanine ammonia lyase (PAL) or chalcone synthase (CHS) enzymes [23,24,25,44]. The phenylpropanoid biosynthetic pathway is upstream of phenolic acid production [45]. Therefore, there may be some *SmKFBs* regulating phenolic acids biosynthesis. Genes having a rather close relationship in the phylogenetic tree may have similar function. Meanwhile, the gene expression pattern is usually related to the gene’s function [46]. Therefore, we used the phylogenetic tree and gene expression pattern to predict the candidate *SmKFB* genes which might involve phenolic acid biosynthesis of *S. miltiorrhiza*. As shown in Figure 1, *SmKFB1* and *SmKFB5* were phylogenetically related to *AtKFB01*, *20*, *39*, and *50*, while *SmKFB28* and *SAGL1* were clustered together with a bootstrap of 100, indicating *SmKFB1*, *5*, and *28* could have similar function in PAL ubiquitination and degradation. In addition, *SmKFB2* and *SmKFB3* were clustered with *AtKFB7* (*AtKFB^CHS^*) in the phylogenetic tree, suggesting that *SmKFB2* and *SmKFB3* may be involved in flavonoid biosynthesis. In general, the gene expression patterns appear to be consistent with their function. Phenolic acids were distributed primarily in the roots and enhanced biosynthesis by MeJA [7,47,48]. According to the gene expression pattern, *SmKFB1* and *SmKFB5* were expressed relatively highly in the root and were strongly suppressed by MeJA (Figure 3b). Therefore, MeJA may suppress gene expression of *SmKFB1* and *SmKFB5* to decrease the degradation of SmPAL and enhance the production of phenolic acids in *S. miltiorrhiza*. Flavonoids are widely distributed throughout the plant kingdom and play an important role in the process of plant physiology. For example, flavonoid functions as a photoprotectant, an ultraviolet ray protectant, and confers flower pigments to attract pollinators. There have been reports that flavonoids were only detected in the aerial parts of *S. miltiorrhiza* [43]. Referring to the gene expression of *SmKFB2,* which showed the lowest expression in the leaf and was suppressed by MeJA, we regard *SmKFB2* as regulating of flavonoid biosynthesis in *S. miltiorrhiza*. Based on the above analysis, *SmKFB1*, *2*, and *5* are most likely involved in phenolic acid biosynthesis. 

## 4. Discussion

*S. miltiorrhiza* is a well-known Chinese herb; tanshinones and phenolic acids are the key secondary metabolites and bioactive compounds of this plant. Thus, to identify the biosynthesis and regulation mechanism of the *S. miltiorrhiza* secondary metabolite is the basis of Danshen quality control. Substantial research has focused on the transcriptional regulation of phenolic acids biosynthesis, but less is known about the protein post-translational modification controlling phenolic biosynthesis. Production of secondary metabolites is influenced greatly by environmental stimuli. Generally, production of secondary metabolites is enhanced when a plant undergoes stresses, which cost energy, photosynthate, and nutrients [49]. This overproduction might cease at the removal of stress. Hence, turning on and turning off the biosynthetic pathway play equally important roles in the plant’s life cycle. As one of protein post-translation modifying manner, the ubiquitin–proteasome system plays an important role in turning off the biosynthetic pathway by mediating the degradation of target proteins such as transcription factors or biosynthetic enzymes. For example, under cold stress, excessive generation of reactive oxygen species (ROS) lead to oxidative damage. Production of proanthocyanins effectively scavenges ROS and facilitates cold tolerance. In apple, *MdMYB23* is a low temperature stable molecule, and activates the transcription of *MdANR,* which promotes biosynthesis of proanthocyanin in apple. On the other hand, *MdMYB23* can be degraded by *MdBT2* mediating UPS, but the gene expression of *MdBT2* is repressed under low temperatures. So *MdMYB23* is stable to regulate *MdANR* gene expression at low temperature. On the contrary, when temperature become suitable, *MdBT2* mediating UPS is activated again and suppresses *MdANR* which, in turn, terminates proanthocyanin biosynthesis [50].

KFB proteins are in charge of the specificity of the SCF E3 Ub ligase complex by selective recruitment of target proteins through the protein–protein interaction domain. KFB proteins are widespread and have been identified for *A. thaliana*, *Vitis vinifera*, *O. sativa*, *P. trichocarpa*, and other plants. To date, for *A. thaliana*, *V. vinifera*, *O. sativa*, and *P. trichocarpa*, 103, 36, 39, and 68 *KFB* genes were identified, respectively [28]. However, we only identified 31 *SmKFB* genes in the *S. miltiorrhiza* genome, which may not represent all KFBs in *S. miltiorrhiza*. One reason for this is the Kelch motif is rather weakly conserved at the sequence level [28]. This is because the Kelch repeat is an ancient motif of 44–56 amino acid residues and is largely defined by the conserved double glycine, tyrosine, tryptophan, and arginine residues. These conserved sites are interspersed with other amino acid residues [51,52]. So, the existence of additional yet undetected *SmKFBs* is likely. Kelch repeats follow a degenerate F-box motif and function as substrate recognition. Individual Kelch motifs form four-stranded *β*-sheets that assemble together to create a *β*-propeller tertiary structure [53,54]. It has been reported that analysis of approximately 400 different plant KFB proteins indicated that 65% of plant KFB proteins contain only one or two Kelch motifs [28]. However, only about 55% (17/31) *SmKFBs* contain less than three Kelch motifs in *S. miltiorrhiza*. In addition, we have found that *SmKFB27* and *SmKFB31* contain four and five Kelch repeats, suggesting they may recognize multiple substrates. 

The biosynthesis and accumulation of secondary metabolites are usually tissue- and developmental stage-specificity [55]. Therefore, the gene expression patterns usually correlate with their function. For example, *OsFBK1*was a F-box Kelch repeat motif protein in rice. Gene expression of *OsFBK1* indicated that it had high transcript abundance in the late anther development stage, and transcript accumulated in increasing order of anther development. Gene function identification of *OsFBK1* showed its involvement in anther cell wall thickenings by mediating turnover of cinnamoyl-CoA reductase in anther [27]. Similarly, *A. thaliana* F-box protein cold temperature-germinating (CTG)-10 is expressed predominantly in hypocotyl, and its overexpression accelerates seed germination [56]. *SmKFB31* transcript level is displayed higher in the flower. As shown in Figure 1, *SmKFB31* is closely related with *AtFBK1* and *OsFBK1*. It has been reported that *AtFBK1* and *OsFBK1* (Os11g34460) functioned as a key regulator in flowering [57], further supporting that *SmKFB31* may be a regulator of flowering of *S. miltiorrhiza*. In addition, *SmKFB5* and *SmKFB25* shared similar expression patterns with higher expression in roots. There is a closely phylogenetic relation of *SmKFB5* with *AtKFB39* and *SmKFB50*, suggesting its function in regulating phenylpropanoid biosynthesis. Furthermore, phenolic acids are also predominately accumulated in roots. Hence, *SmKFB5* could be a candidate gene for studying the mechanism of phenolic acid biosynthesis. In addition, *SmKFB5* is also closely related with *AT3G27150,* which is a responsive gene under phosphate starvation [58]. Our lab has already reported that phosphate starvation promotes the biosynthesis and accumulation of phenolic acids in *S. miltiorrhiza* [59]; however, its detailed molecular mechanism has not been proven. Thus, *SmKFB5* may be a key regulator in mediating phosphate starvation regulating phenolic acid biosynthesis. 

As a key elicitor, JAs have been described to be regulators of a wide array of secondary metabolites, including terpenoids, phenylpropanoids, and alkaloids [60,61]. It is conceivable that JAs might obey diversity regulatory modules to regulate secondary metabolic biosynthesis. Generally, the biosynthesis of tanshinones and phenolic acids were regulated by Jas, usually via controlling the gene expression of JA-responsive transcription factors (such as WRKY, bHLH, MYB, MYC) and biosynthetic pathway genes [9,14,35,62]. However, little is known about the role of MeJA in regulating secondary metabolism on protein post-translational modification level. Some KFB proteins as key subunit of E3 protein ligase have been identified as a regulator in secondary metabolism. In this study, we have found that gene expression of *SmKFB1*, *2*, and *5* was suppressed by MeJA. Those MeJA-repressed *SmKFB* genes are more likely involved in the regulation of tanshinones and phenolic acid biosynthesis, as KFB protein works as a negative regulator to mediate biosynthetic enzyme degradation. In other hand, JAs have been described as positive regulators to promote tanshinones and phenolic acids production [8,48]. Therefore, we speculate that JAs repress *SmKFB* gene expression to decrease the degradation of *SmKFB* targeting biosynthetic enzymes. This might be another molecular mechanism of JAs promoting secondary metabolite biosynthesis.

In future study, we will look into whether MeJA suppresses *SmKFBs* transcripts to maintain the stable of biosynthetic enzyme proteins. If we can illustrate the function of those MeJA-repressed *SmKFBs* in phenolic acid biosynthesis, there will be a new mechanism of JA signal regulating secondary metabolite biosynthesis. 

## 5. Conclusions

In this study, 31 *SmKFB* genes were identified and cloned from the *S. miltiorrhiza* genome. To predict the candidate *SmKFB* genes which may regulate *S. miltiorrhiza* secondary metabolite biosynthesis, phylogenetic tree and gene expression analysis were constructed. Based on these analyses, integration of the phylogenetic tree and gene expression analysis, *SmKFB1*, *2* and *5* were most likely involved in phenolic acid biosynthesis and those functions will be identified in our future study. In addition, this study could be used as a resource for post-translation level regulating secondary metabolite biosynthesis of *S. miltiorrhiza*.

## Figures and Tables

**Table 1:** Sequence features of *SmKFBs* in *S. miltiorrhiza.*

Name	Gene ID	AA len	pI	Mw (Da)	Number of Kelch	Group
*SmKFB1*	MN259124	358	5.16	39075.01	2	II
*SmKFB2*	MN259125	378	4.76	41891.05	1	III
*SmKFB3*	MN259126	372	5.08	40804.4	1	III
*SmKFB4*	MN259127	400	5.86	45334.58	1	III
*SmKFB5*	MN259128	342	5.43	37898.69	3	II
*SmKFB6*	MN259129	388	5.56	43460.15	1	III
*SmKFB7*	MN259130	363	7.47	41147.43	1	III
*SmKFB8*	MN259131	377	9.03	42693.25	1	III
*SmKFB9*	MN259132	372	6.12	41910.27	1	III
*SmKFB10*	MN259133	469	9.72	54239.94	2	III
*SmKFB11*	MN259134	390	8.76	42944.1	2	II
*SmKFB12*	MN259135	397	6.6	44204.99	2	II
*SmKFB13*	MN259136	448	8.75	50471.46	2	III
*SmKFB14*	MN259137	478	6.05	52462.13	3	II
*SmKFB15*	MN259138	404	8.91	45252.13	3	II
*SmKFB16*	MN259139	409	5.39	45846.32	1	III
*SmKFB17*	MN259140	405	5.39	46165.92	1	III
*SmKFB18*	MN259141	450	6.57	50274.37	3	II
*SmKFB19*	MN259142	444	5.66	49289.82	3	II
*SmKFB20*	MN259143	400	6.14	44619.6	3	II
*SmKFB21*	MN259144	395	5.96	43973.2	3	II
*SmKFB22*	MN259145	395	6.12	43520.03	3	II
*SmKFB23*	MN259146	405	8.32	45413.61	3	II
*SmKFB24*	MN259147	365	5.85	41120.89	2	II
*SmKFB25*	MN259148	386	5.86	43279.92	3	II
*SmKFB26*	MN259149	412	9.01	44729.43	1	II
*SmKFB27*	MN259150	458	8.22	50422.57	4	IV
*SmKFB28*	MN259151	439	9.62	49696.45	3	II
*SmKFB29*	MN259152	364	7.59	40315.14	1	I
*SmKFB30*	MN259153	345	6.46	38533.55	3	II
*SmKFB31*	MN259154	476	6.67	52134.89	5	IV

## Supplementary Materials

The following are available online at https://www.mdpi.com/2073-4425/11/5/557/s1. Table S1: List of cloning primers used for *SmKFB* cloning; Table S2: List of sequences used for phylogenetic tree construction; Table S3: List of RT-qPCR primers used for *SmKFB* expression studies; Table S4: *SmKFB* expression data after MeJA treatment.
